# National trends in laryngectomy and the influence of hospital volume on short-term outcomes in Brazil: A 16-year cross-sectional analysis

**DOI:** 10.1016/j.bjorl.2025.101659

**Published:** 2025-08-21

**Authors:** Rafael Filipe Dal Ben Martins, Bruna Andressa Quirino, Leandro Luongo Matos, Marcelo Passos Teivelis, Nelson Wolosker, Ana Kober Nogueira Leite

**Affiliations:** a Hospital Israelita Albert Einstein, São Paulo, SP, Brazil; b Faculdade Israelita de Ciências da Saúde Albert Einstein, Hospital Albert Einstein, São Paulo, SP, Brazil; c Instituto do Câncer do Estado de São Paulo/Hospital das Clínicas da Faculdade de Medicina da Universidade de São Paulo, Departamento de Cirurgia de Cabeça e Pescoço, São Paulo, Brazil; d Faculdade Israelita de Ciências da Saúde Albert Einstein e Instituto de Ensino e Pesquisa Albert Einstein, Hospital Albert Einstein, São Paulo, SP, Brazil

**Keywords:** Larynx, Laryngectomy, Mortality, Laryngeal neoplasms

## Abstract

**Objective:**

Analyze the trends and outcomes of total laryngectomy in Brazil, focusing on the influence of surgical volume on mortality.

**Methods:**

A retrospective, population-based study was conducted using data from the Brazilian Unified Health System (SUS) between 2008 and 2023. Total laryngectomies were identified through specific procedure codes, and hospitals were categorized into quartiles based on surgical volume. The number of procedures over time, mortality rates, and hospital characteristics were analyzed.

**Results:**

A total of 8884 total laryngectomies were performed, decreasing from 1142 in 2008 to 289 in 2023, a 75% reduction (APC = −9.06% per year, p < 0.001). The overall in-hospital mortality rate was 2.7% with no significant change over time Hospitals performing less than two procedures per year had significantly higher mortality rates (3.14%). However, after excluding very low-volume hospitals, mortality rates were similar among the remaining groups.

**Conclusion:**

There was a significant reduction in total laryngectomies in Brazil over the years, suggesting a shift to nonoperative treatment. Moreover, increased mortality in very low-volume hospitals underscores the need for regionalization policies to centralize complex procedures in experienced centers.

## Highlights

• Significant reduction in total laryngectomies in Brazil over 16-years.
• Very low-volume hospitals had significantly higher in-hospital mortality.
• Centralization of laryngectomy services may improve surgical outcomes in Brazil.

## Introduction

Laryngeal cancer is the third most prevalent tumor in the head and neck region, both in Brazil and globally, following only oral cavity and thyroid tumors.^1^ According to the Brazilian National Cancer Institute (INCA), for each year of the 2023–2025 period, approximately 7790 new cases of laryngeal cancer are estimated in Brazil. In terms of mortality, in 2021, laryngeal cancer accounted for 3957 deaths among men (3.3% of total cancer-related deaths in this group) and 602 deaths among women (0.5% of total cancer-related deaths in this group).^2^ Despite its high prevalence, studies in Brazil indicate a decrease in the incidence of laryngeal cancer, although regional disparities persist.^3^, ^4^

For many years, total laryngectomy was the primary treatment method for advanced laryngeal cancer.^5^, ^6^ However, in recent decades, there has been a paradigm shift. In 1991, the Veterans Affairs Laryngeal Cancer Study^7^, ^8^ demonstrated that chemotherapy and radiotherapy are effective alternatives for treating advanced laryngeal cancer. Since then, there has been an increased use of these treatment modalities, leading to a reduction in the number of surgeries performed. This shift has been widely accepted by both physicians and patients due to the stigma associated with total laryngectomy. However, several further studies have already demonstrated that non-surgical treatment may be associated with a worse prognosis.^9^, ^10^

The association between surgical volume and patient outcomes is well established across various surgical specialties, including head and neck surgery.^11^ Several studies have demonstrated that high-volume centers achieve better outcomes when treating head and neck neoplasms.^12^, ^13^ This advantage is particularly significant in high-risk, low-volume procedures, where outcomes can vary considerably between hospitals. Given the recent decline in the indication and frequency of laryngectomies, this phenomenon may be even more pronounced for these procedures.^14^ However, such data remains largely unexplored in Brazil. Therefore, investigating national trends in laryngectomies is essential to understand this scenario better and generate the necessary evidence to inform public health policies. This study aims to analyze trends and outcomes of total laryngectomy in Brazil, focusing on the influence of surgical volume on mortality.

## Methods

This study was a retrospective, quantitative, and descriptive analysis utilizing aggregated populational data. Surgical procedure records were sourced from the publicly accessible database of the Department of Information Technology of the Brazilian Unified Health System (DATASUS),^15^ which is a government-managed digital platform that provides comprehensive data on healthcare procedures performed in Brazil’s public health sector. The analysis covered a period of 16-years, from January 2008 to December 2023.

### Surgical procedures and classifications

The evaluation focused on total laryngectomy procedures, selected according to their respective procedural codes in the SUS database: total laryngectomy (04.04.01.018-0); total laryngectomy with neck dissection in oncology (04.16.13.004-6); total laryngectomy with neck dissection (04.04.01.019-9); and total laryngectomy in oncology (04.16.03.026-2, 04.16.13.005-4).

The study conducted a comprehensive quantitative assessment of multiple variables. Surgical volume was analyzed based on the total number of laryngectomy procedures performed nationwide and across different Brazilian states and hospitals. Hospitalization characteristics were also evaluated, including the average length of hospital stay, the proportion of Intensive Care Unit (ICU) admissions, and the average duration of ICU stays. Mortality rates were examined in terms of in-hospital deaths occurring during the same hospitalization as the laryngectomy. Additionally, healthcare costs were assessed by calculating the average reimbursement per hospitalization and its variation across states. The geographic and demographic distribution of surgical procedures was analyzed by considering surgical volume per 100,000 inhabitants and interstate patient flow, measured as the percentage of surgeries performed outside the patient's municipality of residence.

Hospitals were stratified into quartiles based on the total number of procedures performed. This approach was chosen to classify facilities as high, intermediate, low, or very low volume within the dataset, avoiding reliance on fixed thresholds from existing literature. This method enabled a more context-specific assessment of surgical volume. In this stratification, high-volume hospitals, on average, performed over 11 procedures per year; intermediate-volume hospitals performed between 4.2 and 10 procedures; low-volume hospitals performed between 2 and 4.1 procedures; and very low-volume hospitals performed 2 or fewer procedures.

### Statistical analysis

Trend analysis was conducted using the Joinpoint Regression Program (National Cancer Institute, USA) to assess changes in the number of total laryngectomies performed over time. The Annual Percentage Change (APC) was calculated to quantify the yearly rate of change in procedure volume between 2008 and 2023. A join-point regression analysis was performed to identify potential inflection points where significant trend changes occurred. This method applies a segmented regression approach to detect periods with distinct rates of increase or decline, allowing for a more precise evaluation of temporal variations. The presence of join-points was tested using a Monte Carlo permutation method, and the best-fitting model was selected based on statistical significance (p < 0.05). The APC for each identified segment and 95% Confidence Intervals (95% CIs) were reported.

The Kolmogorov-Smirnov test was performed to assess normality, confirming a non-normal distribution of the data. Therefore, comparative analyses across these quartiles were conducted using the Kruskal-Wallis test, with post-hoc multiple comparisons performed using the Dwass-Steel-Critchlow-Fligner (DSCF) test to identify significant differences in mortality rates, reimbursement per hospitalization, and length of hospital stay across volume categories.

A subgroup analysis investigated whether mortality differences persisted after excluding very low-volume hospitals (those performing fewer than two procedures per year). Facilities were re-stratified into quartiles, and the Kruskal-Wallis test was repeated to evaluate differences among the remaining groups.

## Results

**Fig. 1:**
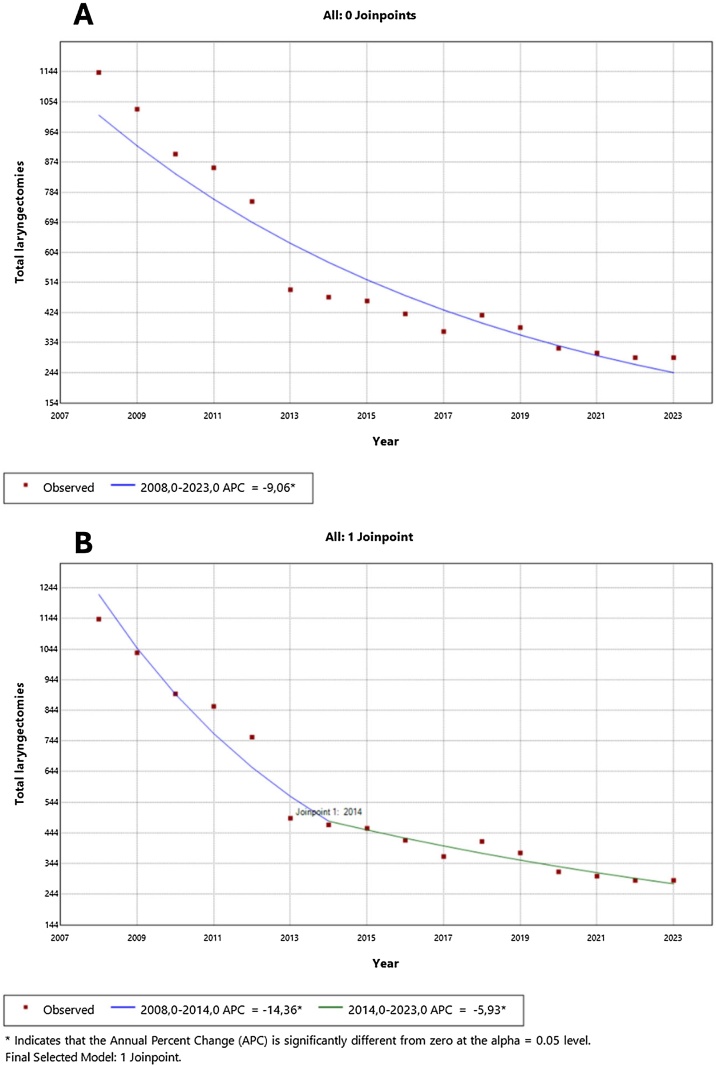
Trends in the number of total laryngectomies performed in the Brazilian Unified Health System (SUS) from 2008 to 2023. (A) Join-point regression analysis without inflection points, showing a single overall declining trend with an Annual Percentage Change (APC) of −9.06% (95% CI: −10.3 to −7.8, p < 0.001). (B) Join-point regression analysis identifying one significant inflection point in 2014. The trend before 2014 showed a steeper decline with an APC of −14.36% (95% CI: −18.1 to −10.4, p < 0.001), while after 2014, the rate of decline slowed to −5.93% (95% CI: −8.2 to −3.6, p < 0.001).

Between January 2008 and December 2023, the Brazilian Unified Health System (SUS) reported a total of 8884 hospitalizations for total laryngectomies (Fig. 1). Among these, 7632 (85.9%) were male, and the average age of the patients was 60.7 years. The in-hospital mortality rate was 2.7%, with no significant changes observed over the years (Annual Percentage Change ‒ APC of 0.13%, p = 0.9). The average length of stay in the hospital was 10.1 days, and 30.1% of these had admissions to Intensive Care Units (ICU), with an average ICU stay of 3.1 days.

The average payment by SUS per hospitalization was BRL 4,541.46, with rates ranging from BRL 2959 in Tocantins to BRL 7,037.38 in Alagoas. On average, 52.9% of the procedures were performed outside the patient’s municipality of residence. In absolute numbers, São Paulo had the highest number of hospitalizations totaling 2982 procedures during this period (an average of 186.4 per year). Rio de Janeiro followed with 1243 hospitalizations (77.7 per year). In contrast, Alagoas registered the lowest number of total laryngectomy hospitalizations, with just 19 during the period (1.2 per year), followed by Roraima with 28 (1.7 per year). Proportionally to the population, Amapá had the highest number of procedures, with 8.3 total laryngectomies per 100,000 inhabitants, followed by Rio de Janeiro (7.2/100,000 inhabitants). In contrast, Alagoas recorded the lowest rate, with only 0.6 laryngectomies per 100,000 inhabitants. The general descriptive data by state are presented in Table 1.

**Table 1:** Descriptive data of laryngectomies performed in the Brazilian Unified Health System (SUS) by Federal Unit from 2008 to 2023.

State	Procedures performed	Procedures/year	Procedures/100.000 inhabitants	Number of deaths	Mortality	Average length of stay	Average length of stay in ICU
Acre	36	2.3	4.1	1	2.8	12.1	2.8
Alagoas	19	1.2	0.6	0	0.0	9.1	3.0
Amapá	67	4.2	8.3	7	10.4	13.6	5.8
Amazonas	191	11.9	4.5	11	5.8	10.4	3.8
Bahia	172	10.8	1.2	4	2.3	7.4	3.3
Ceará	291	18.2	3.2	3	1.0	8.8	2.9
Distrito Federal	158	9.9	5.3	5	3.2	12.7	2.7
Espírito Santo	117	7.3	2.9	0	0.0	6.7	2.4
Goiás	193	12.1	2.6	7	3.6	6.0	2.3
Maranhão	79	4.9	1.1	10	12.7	8.6	4.5
Mato Grosso	88	5.5	2.3	1	1.1	8.1	3.2
Mato Grosso do Sul	48	3.0	1.7	4	8.3	10.6	3.6
Minas Gerais	810	50.6	3.8	14	1.7	8.6	3.2
Pará	151	9.4	1.7	7	4.6	24.6	2.8
Paraíba	111	6.9	2.7	5	4.5	7.7	3.1
Paraná	527	32.9	4.5	13	2.5	6.9	2.2
Pernambuco	284	17.8	3.0	5	1.8	5.2	2.3
Piauí	70	4.4	2.1	2	2.9	6.4	1.4
Rio de Janeiro	1.243	77.7	7.2	26	2.1	10.7	5.6
Rio Grande do Norte	105	6.6	3.0	2	1.9	6.0	2.3
Rio Grande do Sul	581	36.3	5.2	9	1.5	13.0	4.0
Rondônia	52	3.3	3.0	4	7.7	6.4	6.1
Roraima	28	1.8	3.9	0	0.0	11.1	4.6
Santa Catarina	331	20.7	4.1	6	1.8	9.8	2.6
São Paulo	2.982	186.4	6.5	91	3.1	8.4	3.2
Sergipe	77	4.8	3.4	1	1.3	5.9	4.7
Tocantins	73	4.6	4.6	0	0.0	6.8	1.9

Over the years, there has been a significant decline in the total number of laryngectomies performed in Brazil. In 2008, there were 1142 procedures reported, but this number decreased to 289 by 2023. The APC analysis revealed a statistically significant overall decline of −9.06% per year in total laryngectomies performed between 2008 and 2023 (p < 0.001). To further explore the rate of decline over time, a join-point regression analysis was conducted. This method identifies points where significant shifts in trend occur, providing a more precise characterization of temporal variations in the data. The analysis detected a join-point in 2014, indicating a change in the pattern of decline. Before this inflection point (2008–2014), the APC was −14.4% (95% CI: −18.1 to −10.4, p < 0.001), reflecting a steeper reduction in the number of procedures. After 2014 (2014–2023), although the decline continued, it occurred at a slower rate, with an APC of -5.9% (95% CI: −8.2 to −3.6, p < 0.001). These findings suggest that while the total number of laryngectomies in Brazil has consistently decreased, the rate of decline has significantly slowed in the last decade.

During the study period, 238 in-hospital deaths were recorded, resulting in an overall mortality rate of 2.7%. Maranhão had the highest mortality rate (12.7%), followed by Amapá (10.4%). Conversely, the states of Alagoas, Espírito Santo, Roraima, and Tocantins reported no in-hospital deaths during the period.

The procedures were conducted in 365 facilities, with just 8 (2.2%) accounting for 25% of all procedures. Only seven facilities (1.9%) performed more than one procedure per month on average. Approximately 25.8% of the procedures were conducted in facilities performing fewer than two procedures per year on average, and 69.3% of the facilities performed fewer than one procedure per year on average.

Facilities were stratified into quartiles based on the total number of procedures, with each quartile representing 25% of the total performed. These quartiles were used to classify facilities as high, intermediate, low, or very low volume. Comparative analysis among quartiles revealed significant differences in studied variables across high, intermediate, low, and very low-volume facilities (Kruskal-Wallis test). Key differences were observed in mortality rate (p < 0.001), mean payment per hospitalization (p = 0.01), and average length of stay (p = 0.002). Post-hoc analysis using the Dwass-Steel-Critchlow-Fligner test identified significant differences in mortality rates between all groups and the very low-volume group, establishing this group as the highest risk category (Table 2).

**Table 2:** Comparison of in-hospital mortality, costs, length of stay, and ICU utilization across facility volume groups.

Group	High volume (HV)	Intermediate volume (IV)	Low volume (LV)	Very low volume (VLV)	p (Kruskal-wallis)	DSCF multiple comparisons
Hospitalizations/year	17.18 ± 8.21	6.58 ± 1.96	3.11 ± 0.68	0.48 ± 0.47	‒	‒
Average cost per hospitalization	3,919.2 ± 1,210.2	4,668.8 ± 1,022.5	4,924.5 ± 1,122.0	3,928.7 ± 2,483.5	0.009	HV × IV p = 0.521
						HV × LV p = 0.120
						HV × VLV p = 0.997
						IV × LV p = 0.668
						IV × VLV p = 0.354
						**LV** × **VLV p = 0.014**
Mortality rate (%)	2.28 ± 1.67	2.53 ± 2.46	2.59 ± 3.22	3.14 ± 11.74	<0.001	HV × IV p = 0.972
						HV × LV p = 0.972
						**HV** × **VLV p ≤ 0.001**
						IV × LV p = 0.959
						**IV** × **VLV p ≤ 0.001**
						**LV** × **VLV p ≤ 0.001**
Average length of stay	9.12 ± 3.85	11.24 ± 5.04	10.10 ± 3.72	8.84 ± 7.73	0.002	HV × IV p = 0.816
						HV × LV p = 0.993
						HV × VLV p = 0.835
						IV × LV p = 0.914
						**IV** × **VLV p = 0.030**
						**LV** × **VLV p = 0.015**
Percentage of Hospitalizations with use of ICU	4.15 ± 3.15	4.36 ± 2.83	2.74 ± 2.10	2.48 ± 3.28	<0.001	HV × IV p = 0.937
						HV × LV p = 0.584
						HV × VLV p = 0.196
						IV × LV p = 0.077
						**IV** × **VLV p = 0.001**
						LV × VLV p = 0.112

After establishing that the very low-volume group exhibited significantly higher mortality, a subgroup analysis was performed excluding this group. The remaining facilities were re-divided into quartiles to determine whether the risk persisted among low-volume establishments. After excluding very low-volume facilities (defined as those performing fewer than approximately two procedures per year), no significant differences in mortality were observed among the new quartiles (Kruskal-Wallis test; p = 0.22).

## Discussion

The Brazilian healthcare system operates under a mixed model, integrating public and private sectors. Approximately one-quarter of the population has access to private healthcare services, and most individuals rely solely on the publicly funded Unified Health System (SUS).^16^ This study analyzed exclusively data from SUS. Our findings confirmed that hospitals performing a low volume of surgeries were associated with significantly higher mortality rates, reinforcing the established volume-outcome relationship in surgical oncology. To our knowledge, this association had not been previously documented in Brazil for laryngectomies.

Over the study period, the number of total laryngectomies performed in the SUS decreased by approximately 75%, a trend consistent with global data.^17^, ^18^ This decline is likely related to the increased indication of non-surgical treatments, particularly chemoradiotherapy, following landmark studies.^7^, ^8^ Join-point regression analysis identified a more pronounced decline between 2008 and 2014, with a steeper APC coinciding with the widespread adoption of chemoradiotherapy as the preferred treatment modality for advanced laryngeal cancer. However, this decline has slowed in recent years, suggesting that the proportion of patients undergoing total laryngectomy is stabilizing. While this shift offers functional benefits for selected patients, concerns remain regarding oncologic outcomes, especially in low-resource settings where access to advanced radiotherapy is limited.^19^ To fully understand the magnitude of this decline, it is essential to analyze the epidemiology of laryngeal cancer in Brazil.

Studies indicate a decreasing incidence of laryngeal cancer both globally and in Brazil,^3^, ^4^ likely associated with reduced tobacco consumption. However, while significant, the decline in incidence does not fully account for the drastic reduction in total laryngectomies. Fonte et al. reported a decrease in the incidence rate from 9.2 to 4.5 per 100,000 men (APC = −3.1%) and from 1.26 to 0.48 per 100,000 women (APC = −3.0%) between 2000 and 2019. Conversely, our study found an APC of −9.06% for total laryngectomies, indicating that the incidence decline alone cannot justify the observed reduction.

Given that total laryngectomy is primarily indicated for advanced laryngeal tumors, one hypothesis is that a reduction in advanced-stage cases due to earlier diagnosis could explain the decline. However, a study evaluating hospital-based cancer registries demonstrated that the proportion of advanced-stage laryngeal cancers in Brazil has remained stable at approximately 60% of diagnosed cases.^20^ This suggests that the decrease in laryngectomies is more likely driven by a growing preference for nonsurgical treatment with chemoradiotherapy, which aligns with international trends. This is further supported by data indicating a significant increase in the use of chemotherapy for laryngeal cancer in the SUS starting in 2007.^4^ Additionally, mortality rates have remained stable despite the decreasing incidence, with some authors projecting a future increase,^21^ raising concerns about oncologic outcomes following nonsurgical treatment. Previous evidence suggests that chemoradiotherapy may result in lower overall survival compared to surgery, with potential implications for cancer-related mortality.^19^ Furthermore, our study confirms, using Brazilian data, the association between hospital volume and in-hospital mortality following total laryngectomy. Hospitals performing fewer than two laryngectomies per year exhibited significantly higher mortality rates. This finding aligns with previous research demonstrating that high-volume centers achieve superior surgical outcomes due to better perioperative management, multidisciplinary expertise, and adherence to best practices.^13^, ^22^ Similar findings have been consistently reported in other oncologic surgeries, including colorectal, gastric, and pancreatic cancer resections.^23^, ^24^ Although our analysis was limited to hospital volume, previous studies have shown that surgeon volume accounts for up to 95% of the observed hospital volume effect on mortality.^13^

The impact of hospital volume on mortality was further highlighted by our subgroup analysis, which excluded very low-volume hospitals. After removing these facilities from consideration, the mortality differences among the remaining quartiles became not statistically significant. This suggests the existence of a critical volume threshold below which surgical outcomes are significantly compromised, which aligns with previous findings in head and neck cancer surgery.^25^

The current landscape of laryngeal cancer in Brazil raises critical concerns. Although the decreasing incidence of laryngeal cancer has not translated into a reduction in mortality, emphasizing the need for individualized treatment decisions based on multidisciplinary evaluation.^26^ Non-surgical treatment should not be indiscriminately recommended but rather carefully selected, ideally in specialized centers with expertise in head and neck oncology.

The substantial decline in total laryngectomies has significant implications for surgical training. With fewer procedures being performed, future head and neck surgeons may have limited experience managing these cases. The decreasing number of specialists and institutions performing laryngectomies, combined with the growing proportion of salvage surgeries following failed chemoradiotherapy, is likely to increase surgical complexity, complication rates, and adverse outcomes. In this context, structured referral pathways and centralized care models for laryngeal cancer treatment are urgently needed.

Currently, 25% of total laryngectomies in Brazil are performed in very low-volume hospitals, a proportion that may continue to rise. Our findings underscore the need for regionalizing laryngectomy services to concentrate laryngeal cancer care in experienced centers. Previous initiatives, such as the Volume Pledge adopted by leading U.S. institutions, have demonstrated that setting minimum volume standards for high-risk surgeries can improve patient outcomes.^13^ Brazil may benefit from similar public policies, such as integrating specialist training programs and incentivizing referral networks to optimize surgical oncology care.^5^ Beyond reducing surgical complications, centralization could also enhance individualized decision-making regarding treatment selection.

However, it is important to recognize that surgical quality is influenced by multiple factors beyond volume alone. Some experts argue against using volume as the sole criterion for patient care and advocate for further studies to better determine its actual impact.^27^, ^28^ The inability to explore these additional variables represents a potential limitation of our study.

Additional limitations of this study are related to constraints of publicly available datasets. Specifically, data on the presence of specialized physicians within institutions, surgeon qualifications, and postoperative complications other than in-hospital mortality were unavailable. Furthermore, our analysis was limited to the public healthcare sector, excluding data from private institutions.

## Conclusion

This study demonstrates a significant decline in total laryngectomies in Brazil, exceeding the reduction in laryngeal cancer incidence. This suggests a shift towards the use of non-surgical treatments. However, the stable rates of advanced-stage disease raise concerns about oncologic outcomes, particularly in resource-limited settings.

Our findings confirm that very low-volume centers (fewer than 2 procedures per year) are associated with higher mortality rates. This reinforces the need for regionalized referral systems to ensure that complex surgeries are performed in experienced centers. While volume influences outcomes, it should not be the sole criterion for determining care allocation. Furthermore, additional research is needed to identify additional quality metrics in surgical oncology.

## ORCID

Rafael Filipe Dal Ben Martins: 0009-0005-3190-658X

Bruna Andressa Quirino: 0009-0008-4694-0678

Leandro Luongo Matos: 0000-0002-5068-8208

Marcelo Passos Teivelis: 0000-0002-3648-6773

Nelson Wolosker: 0000-0003-1991-3507

Ana Kober Nogueira Leite: 0000-0001-7814-757X

## Funding

No external funding was received for this research.

## Declaration of competing interest

The authors declare no conflicts of interest.

## References

[1] Bray F., Ferlay J., Soerjomataram I., Siegel R.L., Torre L.A., Jemal A. (2018). Global cancer statistics 2018: GLOBOCAN estimates of incidence and mortality worldwide for 36 cancers in 185 countries. CA Cancer J Clin.

[2] Instituto Nacional de Cancer. Estimativa 2023: incidência de câncer no Brasil. Accessed Novembro 2023, https://www.inca.gov.br/sites/ufu.sti.inca.local/files//media/document//estimativa-2023.pdf.

[3] Viana L.P., Bustamante-Teixeira M.T., Malta D.C. (2022). Trend of the Burden of Larynx Cancer in Brazil, 1990 to 2019. Rev Soc Bras Med Trop.

[4] da Fonte A.L.F., Costa G.J., da Fonte Neto A.S., Pinto R.A., de Mello M.J.G. (2023). Epidemiology of laryngeal cancer in Brazil: historical data from 2000 to 2019. Cancer Epidemiol.

[5] Babin E., Blanchard D., Hitier M. (2011). Management of total laryngectomy patients over time: from the consultation announcing the diagnosis to long term follow-up. Eur Arch Otorhinolaryngol.

[6] Chen A.Y., Schrag N., Hao Y. (2006). Changes in treatment of advanced laryngeal cancer 1985-2001. Otolaryngol Head Neck Surg.

[7] Wolf G.T., Fisher S.G., Department of Veterans Affairs Laryngeal Cancer Study Group (1991). Induction chemotherapy plus radiation compared with surgery plus radiation in patients with advanced laryngeal cancer. N Engl J Med.

[8] Forastiere A.A., Goepfert H., Maor M. (2003). Concurrent chemotherapy and radiotherapy for organ preservation in advanced laryngeal cancer. N Engl J Med.

[9] Hoffman H.T., Porter K., Karnell L.H. (2006). Laryngeal cancer in the United States: changes in demographics, patterns of care, and survival. Laryngoscope.

[10] Calvas O.I.J., Ramos D.M., Matos L.L. (2017). Oncological results of surgical treatment versus organ-function preservation in larynx and hypopharynx cancer. Rev Assoc Med Bras (1992).

[11] Luft H.S., Bunker J.P., Enthoven A.C. (1979). Should operations be regionalized? The empirical relation between surgical volume and mortality. N Engl J Med.

[12] Gourin C.G., Frick K.D. (2012). National trends in laryngeal cancer surgery and the effect of surgeon and hospital volume on short-term outcomes and cost of care. Laryngoscope.

[13] Saraswathula A., Austin J.M., Fakhry C. (2023). Surgeon volume and laryngectomy outcomes. Laryngoscope.

[14] Maddox P.T., Davies L. (2012). Trends in total laryngectomy in the era of organ preservation: a population-based study. Otolaryngol Head Neck Surg.

[15] Saúde Md. Tabnet DATASUS. Accessed October, 2024.

[16] (IBGE) IBdGeE. Pesquisa Nacional de Saúde 2019: Indicadores de saúde e mercado de trabalho 2020.

[17] Verma S.P., Mahboubi H. (2014). The changing landscape of total laryngectomy surgery. Otolaryngol Head Neck Surg.

[18] Rzepakowska A., Żurek M., Niemczyk K. (2021). Review of recent treatment trends of laryngeal cancer in Poland: a population-based study. BMJ Open.

[19] Pfuetzenreiter E.G., Ferreron G.F., Sadka J.Z. (2024). Total laryngectomy vs. non-surgical organ preservation in advanced laryngeal cancer: a metanalysis. Braz J Otorhinolaryngol.

[20] Nascimento de Carvalho F., de Camargo Cancela M., Mesentier da Costa L. (2025). Disparities in stage at diagnosis of head and neck tumours in Brazil: a comprehensive analysis of hospital-based cancer registries. Lancet Reg Health Am.

[21] Dantas de Oliveira N.P., Barbosa I.R., Vieria Paulino J.N., de Camargo Cancela M., Bezerra de Souza D.L. (2016). Regional and gender differences in laryngeal cancer mortality: trends and predictions until 2030 in Brazil. Oral Surg Oral Med Oral Pathol Oral Radiol.

[22] Gourin C.G., Stewart C.M., Frick K.D. (2019). Association of hospital volume with laryngectomy outcomes in patients with larynx cancer. JAMA Otolaryngol Head Neck Surg.

[23] Tustumi F., Portilho A.S., Teivelis M.P. (2023). The impact of the institutional abdominoperineal resections volume on short-term outcomes and expenses: a nationwide study. Tech Coloproctol.

[24] Yeh C.M., Lai T.Y., Hu Y.W., Teng C.J., Huang N., Liu C.J. (2024). The impact of surgical volume on outcomes in newly diagnosed colorectal cancer patients receiving definitive surgeries. Sci Rep.

[25] Kowalski L.P. (2023). Eugene Nicholas Myers’ lecture on head and neck cancer, 2020: the surgeon as a prognostic factor in head and neck cancer patients undergoing surgery. Int Arch Otorhinolaryngol.

[26] Cernea C.R., Leite A.K., Muller B.F., Matos L.L. (2024). Multidisciplinary approach in head and neck cancer. Einstein (Sao Paulo).

[27] Heiden B.T., Kozower B.D. (2022). Keeping a safe distance from surgical volume standards. J Clin Oncol.

[28] Livingston E.H., Cao J. (2010). Procedure volume as a predictor of surgical outcomes. JAMA.

